# Idiopathic Hypereosinophilic Syndrome Presenting as Isolated Right Ventricular Endomyocardial Fibrosis With Intracavitary Thrombus: A Multimodality Imaging Case Report

**DOI:** 10.7759/cureus.113350

**Published:** 2026-07-25

**Authors:** Nadia Loudiyi, Mohamed Malki, Hafsa Erregui, Najat Mouine, Aatif Benyass

**Affiliations:** 1 Cardiology, Mohammed V Military Teaching Hospital, Rabat, MAR

**Keywords:** cardiac magnetic resonance, endomyocardial fibrosis, hypereosinophilic syndrome, intracavitary thrombus, multimodality imaging, right ventricle

## Abstract

Idiopathic hypereosinophilic syndrome (HES) is an uncommon disorder characterized by persistent eosinophilia and eosinophil-mediated organ damage, with cardiac involvement representing its most severe complication. Isolated right ventricular endomyocardial fibrosis is exceptionally uncommon and may mimic other intracardiac pathologies, making diagnosis particularly challenging. We report the case of a 55-year-old man who presented with a one-month history of progressive bilateral lower-limb edema. Laboratory investigations revealed marked persistent hypereosinophilia associated with elevated cardiac biomarkers. Transthoracic echocardiography demonstrated an extensive right ventricular intracavitary mass with near-complete apical obliteration. Cardiac magnetic resonance confirmed diffuse right ventricular subendocardial late gadolinium enhancement consistent with endomyocardial fibrosis and identified an associated intracavitary thrombus. CT excluded pulmonary embolism and malignancy while confirming the intracardiac lesion. An extensive infectious, autoimmune, hematologic, molecular, and oncologic evaluation, including bone marrow examination and molecular testing for clonal eosinophilic disorders, was negative, leading to the diagnosis of idiopathic HES. Treatment with high-dose corticosteroids, vitamin K antagonist anticoagulation, and diuretic therapy was initiated. This case highlights the diagnostic challenge posed by isolated right ventricular eosinophilic endomyocardial fibrosis and underscores the complementary role of multimodality cardiovascular imaging in lesion characterization, exclusion of differential diagnoses, and guidance of appropriate management. Early recognition and a systematic etiological workup are essential to prevent irreversible cardiac damage and improve patient outcomes.

## Introduction

Hypereosinophilic syndrome (HES) is a rare and heterogeneous disorder characterized by persistent eosinophilia associated with eosinophil-mediated organ damage [1]. Cardiac involvement represents its most severe manifestation and remains the leading cause of morbidity and mortality, with eosinophilic heart disease typically progressing through three overlapping stages: acute myocardial injury, intracardiac thrombosis, and chronic endomyocardial fibrosis [2,3]. Early recognition is crucial because timely treatment may prevent irreversible myocardial damage and improve clinical outcomes [3,4].

Because idiopathic HES is a diagnosis of exclusion, a comprehensive evaluation is required to rule out secondary, infectious, autoimmune, neoplastic, and clonal causes of eosinophilia [1,4]. Isolated right ventricular endomyocardial fibrosis is an exceptionally rare manifestation of eosinophilic heart disease and may mimic other intracardiac masses, making diagnosis particularly challenging [5-7]. We report a rare case of idiopathic HES presenting with isolated right ventricular endomyocardial fibrosis and an associated intracavitary thrombus, highlighting the pivotal role of multimodality cardiovascular imaging and a systematic etiological workup in establishing an accurate diagnosis and guiding appropriate management.

## Case presentation

A 55-year-old man with no cardiovascular risk factors and no significant past medical history was admitted to our institution because of progressively worsening bilateral lower-limb edema that had been evolving over the preceding month. He denied exertional dyspnea, chest pain, palpitations, syncope, fever, weight loss, night sweats, asthma, allergic disease, recent travel, exposure to parasites, or recent medication use.

On admission, the patient was conscious, alert, and in no acute respiratory distress. He was hemodynamically stable, with a blood pressure of 120/80 mmHg, a regular heart rate of 80 beats/min, and an oxygen saturation of 98% on room air. He was afebrile and showed no signs of peripheral hypoperfusion. Physical examination revealed bilateral, symmetrical, pitting lower-limb edema extending to the legs, associated with elevated jugular venous pressure, supporting a cardiac origin and suggesting right-sided heart failure. There was no calf tenderness, erythema, or asymmetry suggestive of deep venous thrombosis. Cardiac auscultation revealed regular heart sounds and a grade 3/6 holosystolic murmur at the left lower sternal border, consistent with tricuspid regurgitation, without gallop rhythm or pericardial rub. Pulmonary examination showed normal respiratory effort, preserved vesicular breath sounds bilaterally, and no wheezing or crackles; there were no clinical signs of pulmonary congestion or bronchospasm. The abdomen was soft and non-tender, with no clinically evident hepatomegaly, ascites, or hepatojugular reflux. There was no lymphadenopathy, skin rash, purpura, livedo reticularis, digital ischemia, or other peripheral manifestations suggestive of systemic embolization, vasculitis, allergic disease, or connective tissue disorder.

**Figure 1:**
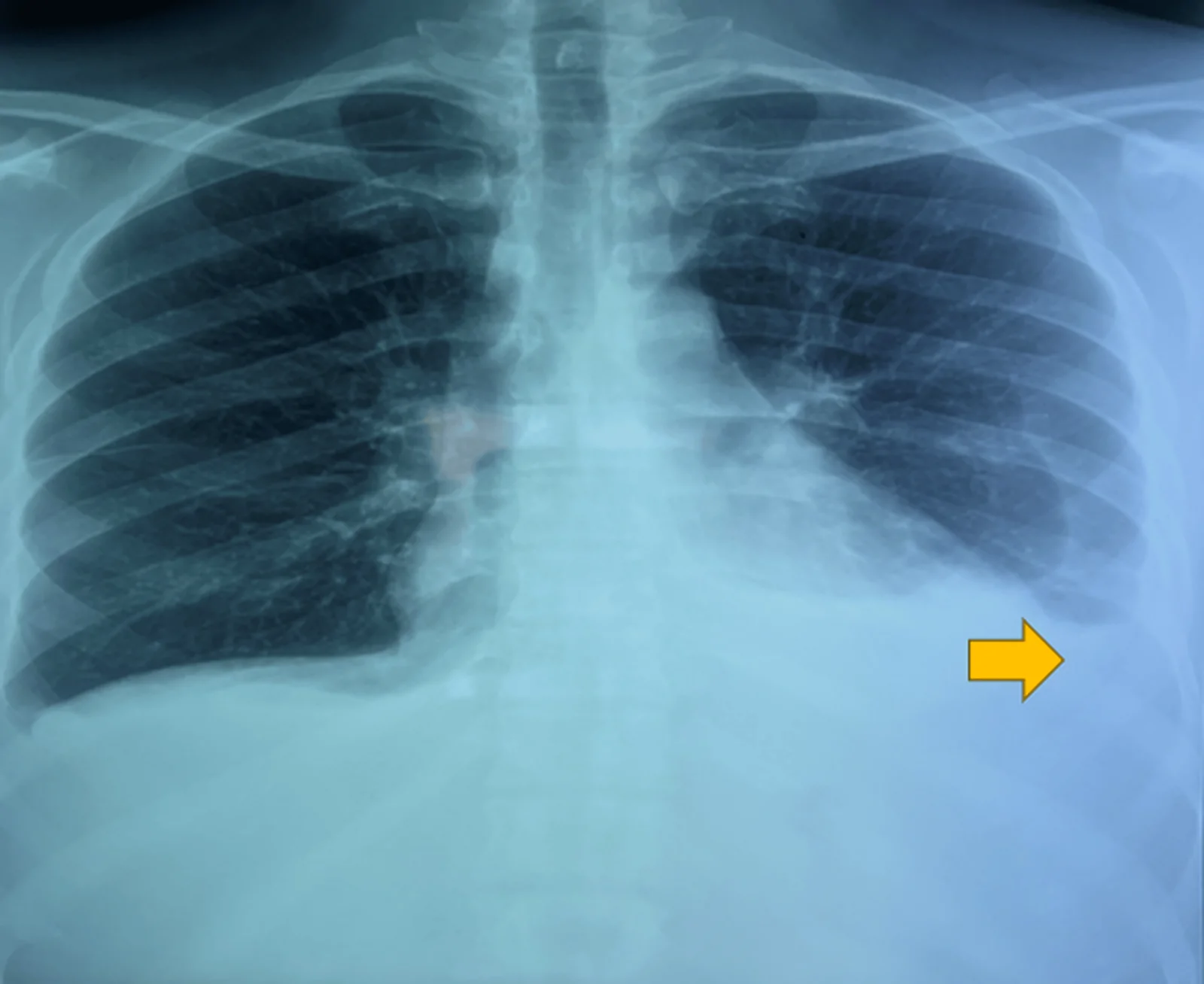
Chest radiograph at presentation. Chest radiograph demonstrating a normal cardiac silhouette without cardiomegaly and a left-sided pleural effusion (yellow arrow).

Electrocardiography demonstrated normal sinus rhythm without conduction abnormalities, ischemic changes, or ventricular hypertrophy. Chest radiography showed a normal cardiac silhouette without cardiomegaly and a left-sided pleural effusion (Figure 1).

Initial laboratory investigations demonstrated leukocytosis with marked hypereosinophilia associated with elevated cardiac biomarkers, including high-sensitivity cardiac troponin and N-terminal pro-B-type natriuretic peptide, while the remaining hematological and biochemical parameters were within normal limits (Table 1). To confirm the persistence of the marked eosinophilia and exclude a transient or laboratory-related abnormality, the complete blood count was repeated the following day, demonstrating persistent hypereosinophilia with a comparable absolute eosinophil count, thereby supporting the diagnosis of true hypereosinophilia.

**Table 1:** Baseline laboratory investigations at hospital admission. Baseline laboratory findings obtained at admission. Marked hypereosinophilia was the predominant abnormality, accompanied by leukocytosis and elevated cardiac biomarkers (high-sensitivity cardiac troponin and NT-proBNP), whereas inflammatory markers, renal function, liver function tests, coagulation profile, and D-dimer were within the normal reference ranges. ALT, alanine aminotransferase; AST, aspartate aminotransferase; eGFR, estimated glomerular filtration rate; LDH, lactate dehydrogenase; NT-proBNP, N-terminal pro-B-type natriuretic peptide; PT, prothrombin time; TSH, thyroid-stimulating hormone

Parameter	Patient value	Reference range
Hemoglobin	14 g/dL	13.0-17.0 g/dL
White blood cell count	17.2 × 10⁹/L	4.0-10.0 × 10⁹/L
Neutrophils	5.2 × 10⁹/L	2.0-7.5 × 10⁹/L
Lymphocytes	2.3 × 10⁹/L	1.0-4.0 × 10⁹/L
Eosinophils	9.0 × 10⁹/L	0.02-0.50 × 10⁹/L
Basophils	0.1 × 10⁹/L	0.00-0.10 × 10⁹/L
Platelets	200 × 10⁹/L	150-400 × 10⁹/L
PT	100%	70-100%
D-dimer	0.30 mg/L FEU	<0.50 mg/L FEU
Sodium	135 mmol/L	135-145 mmol/L
Potassium	4.5 mmol/L	3.5-5.1 mmol/L
Urea	0.32 g/L	0.15-0.45 g/L
Creatinine	8 mg/L	6-12 mg/L
eGFR	70 mL/min/1.73 m²	≥60 mL/min/1.73 m²
AST	30 U/L	<40 U/L
ALT	30 U/L	<41 U/L
Ferritin	100 ng/mL	30-400 ng/mL
LDH	350 U/L	<480 U/L
NT-proBNP	3,000 pg/mL	<125 pg/mL
High-sensitivity cardiac troponin	284 ng/L	<14 ng/L
C-reactive protein	3 mg/L	<5 mg/L
TSH	0.7 mIU/L	0.4-4.0 mIU/L

**Figure 2:**
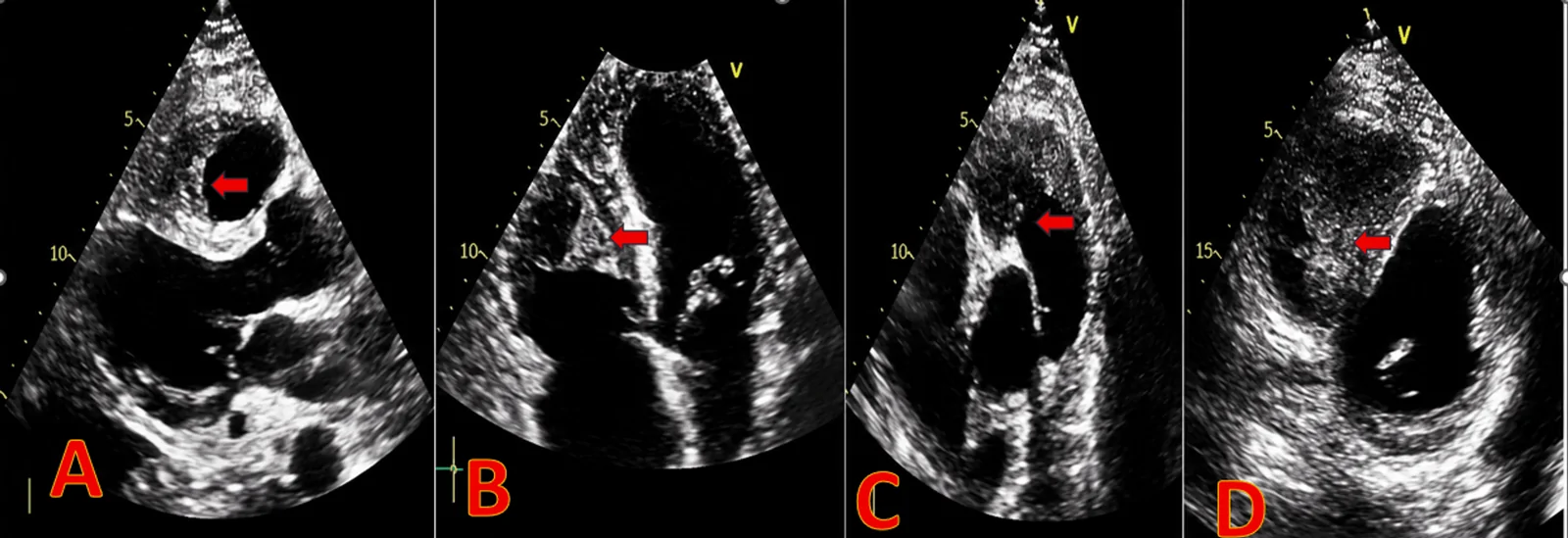
Transthoracic echocardiographic images. (A-D) Multiple views demonstrating an echogenic intracavitary right ventricular mass (red arrows), extending to the right ventricular outflow tract (pulmonary infundibulum) and attached to the septal leaflet of the tricuspid valve.

Transthoracic echocardiography revealed a non-dilated, non-hypertrophied left ventricle with preserved systolic function and no regional wall motion abnormalities (Figure 2). The right ventricle was not dilated and had preserved systolic function. A large homogeneous echogenic mass extensively occupied the right ventricular cavity, extending from the apex to the right ventricular outflow tract and involving the septal leaflet of the tricuspid valve, resulting in near-complete obliteration of the right ventricular apex. The right atrium was markedly dilated and free of spontaneous echo contrast or thrombus. Doppler examination demonstrated moderate tricuspid regurgitation, an estimated pulmonary artery systolic pressure of 40 mmHg, a dilated but collapsible inferior vena cava, and no pericardial effusion.

**Figure 3:**
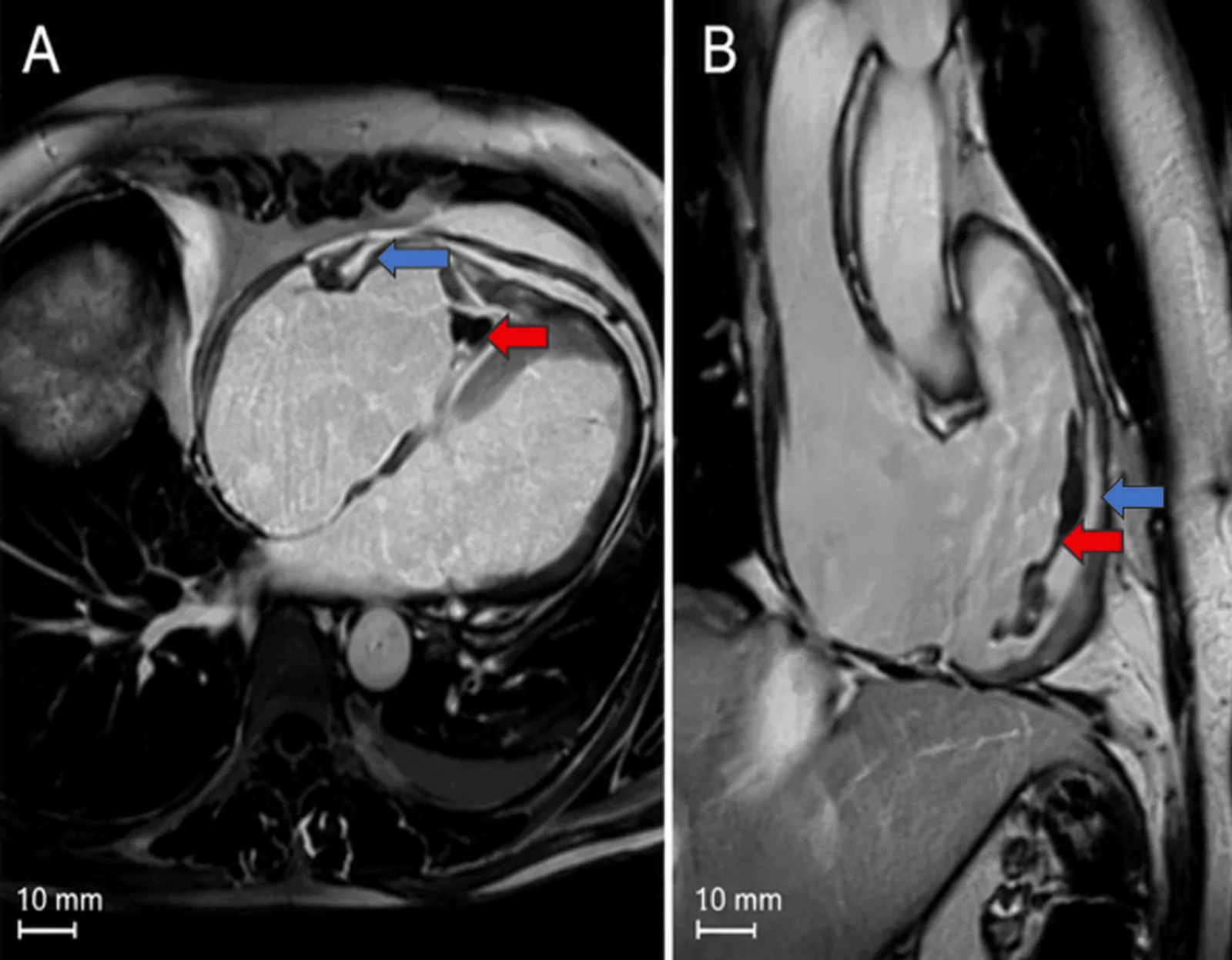
CMR LGE imaging demonstrating isolated right ventricular endomyocardial fibrosis with associated apical thrombus. (A) Apical four-chamber LGE image and (B) right ventricular inflow-outflow LGE image demonstrating diffuse circumferential subendocardial LGE of the right ventricle (blue arrows), consistent with right ventricular endomyocardial fibrosis, associated with an apical intracavitary thrombus (red arrows). CMR, cardiac magnetic resonance; LGE, late gadolinium enhancement

**Figure 4:**
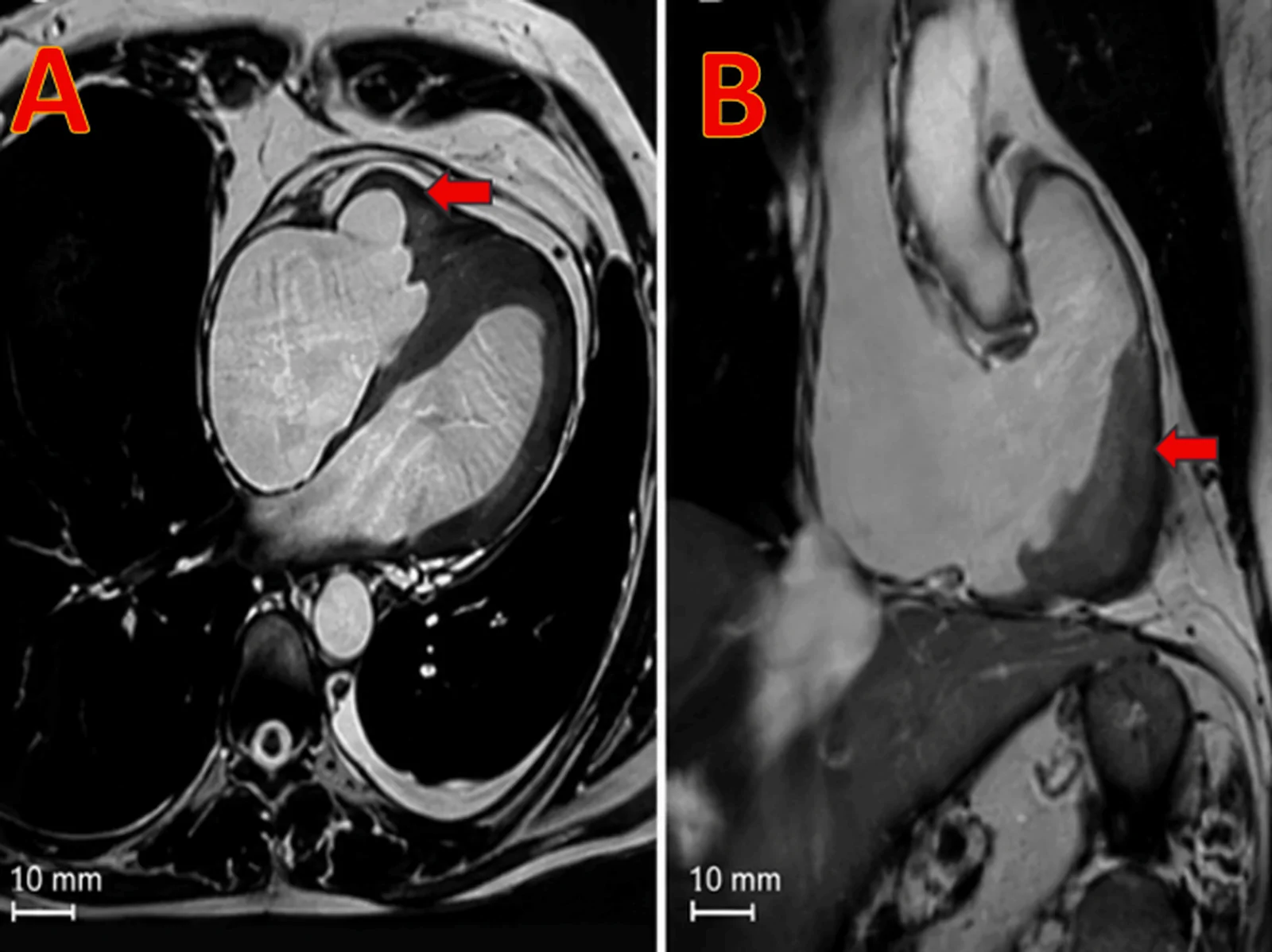
Cine steady-state free precession CMR images demonstrating isolated right ventricular endomyocardial fibrosis. (A) Apical four-chamber view and (B) right ventricular inflow-outflow view demonstrating extensive fibrotic obliteration of the right ventricular apex and inflow tract (red arrows), resulting in partial obliteration and severe reduction of the right ventricular cavity, consistent with advanced right ventricular endomyocardial fibrosis. CMR, cardiac magnetic resonance

Because of the unusual appearance of the intracardiac lesion, cardiac MRI was performed for tissue characterization (Figure 3, 4). Cine steady-state free precession sequences demonstrated marked obliteration of the right ventricular apex with extension toward the inflow tract. Late gadolinium enhancement (LGE) sequences showed diffuse circumferential subendocardial enhancement involving the right ventricular apex and inflow tract, consistent with endomyocardial fibrosis. A right ventricular apical intracavitary thrombus was also identified.

**Figure 5:**
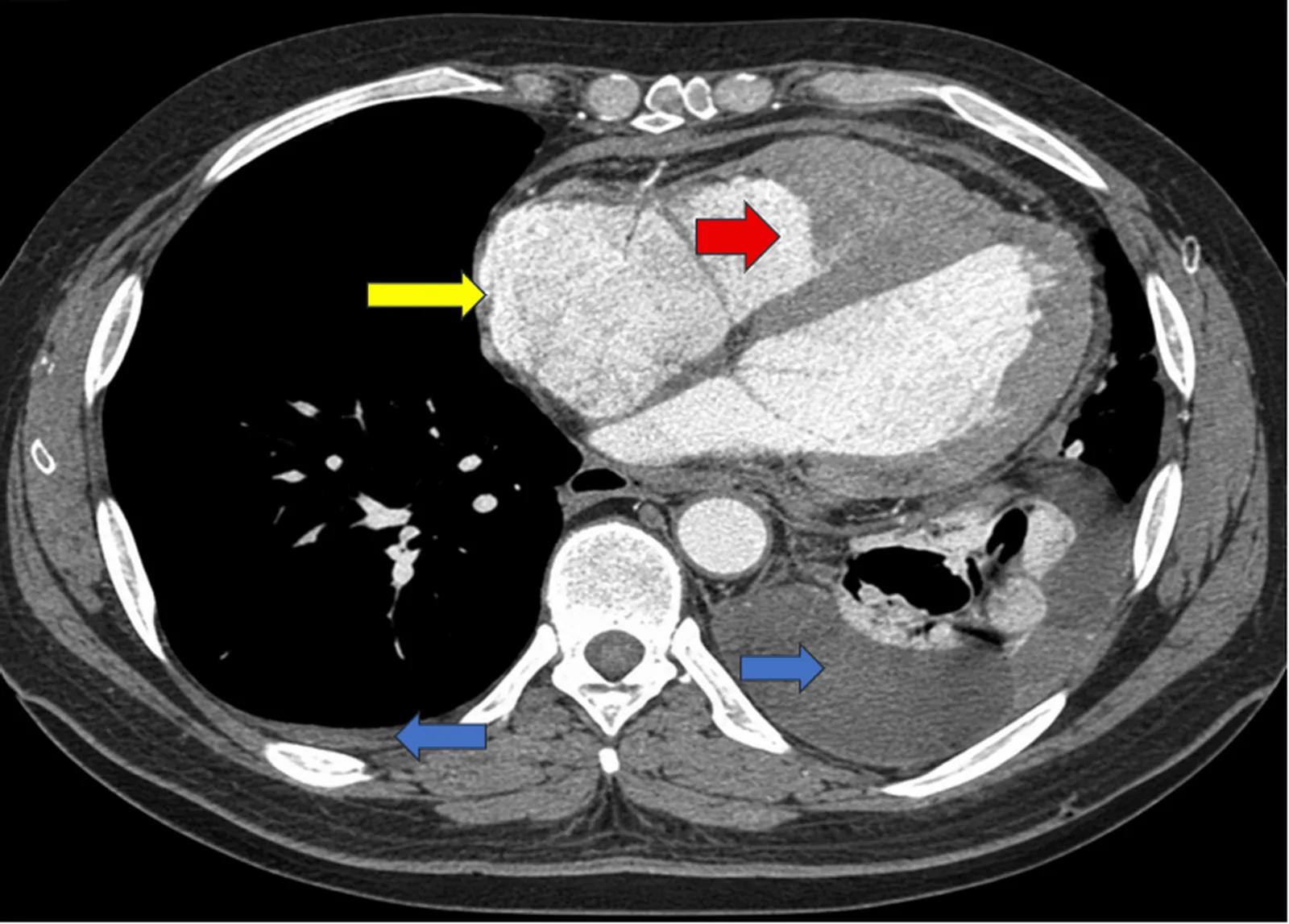
Contrast-enhanced CT of the chest. Axial contrast-enhanced CT image demonstrating a right ventricular intracavitary mass (red arrow) associated with right atrial dilatation (yellow arrow). Bilateral pleural effusions are also present (blue arrows), more prominent on the left side.

CT pulmonary angiography confirmed marked right atrial enlargement and a large filling defect occupying the right ventricular cavity, corresponding to the lesion identified on echocardiography and cardiac MRI (Figure 5). Bilateral pleural effusions, more pronounced on the left side, were present without evidence of pulmonary embolism. In addition, contrast-enhanced thoracoabdominopelvic CT, performed to investigate an underlying malignancy, revealed no mediastinal, hilar, abdominal, or pelvic lymphadenopathy and no evidence of a primary or metastatic malignancy.

Diagnostic thoracentesis yielded a citrine-yellow transudative pleural effusion with a protein concentration of 20 g/L and a lactate dehydrogenase level of 100 U/L. Gram staining and bacterial cultures were negative, and cytological examination revealed no malignant cells.

Given the marked eosinophilia associated with cardiac involvement, a comprehensive etiological workup was undertaken. Infectious investigations, including stool examinations for ova and parasites (three samples), parasitic serologies (*Strongyloides*, *Toxocara*, *Schistosoma*, and *Trichinella*), serological testing for human immunodeficiency virus, hepatitis B virus, and hepatitis C virus, as well as polymerase chain reaction testing for SARS-CoV-2, respiratory syncytial virus, influenza A, and influenza B viruses, were all negative.

Autoimmune investigations, including antinuclear antibodies, antineutrophil cytoplasmic antibodies, rheumatoid factor, antiphospholipid antibodies, and complement fractions, were unremarkable. Complement levels were within the normal range (C3: 1.20 g/L, reference range 0.90-1.80 g/L; C4: 0.28 g/L, reference range 0.10-0.40 g/L). Total serum immunoglobulin E was also within the normal range (45 IU/mL; reference range <100 IU/mL), making an allergic etiology unlikely.

A comprehensive hematologic evaluation was subsequently performed. Bone marrow aspiration and biopsy demonstrated eosinophilic hyperplasia without blasts or evidence of hematologic malignancy. Flow cytometric immunophenotyping did not identify an abnormal lymphoid population. Molecular analyses for FIP1L1-PDGFRA, PDGFRB, FGFR1, JAK2 V617F, CALR, MPL, and BCR::ABL1 were all negative. Serum protein electrophoresis revealed no monoclonal gammopathy. Tumor markers, including carcinoembryonic antigen, carbohydrate antigen 19-9, carbohydrate antigen 125, alpha-fetoprotein, prostate-specific antigen, and β-human chorionic gonadotropin, were all within the normal reference range.

Following multidisciplinary discussion between the cardiology and internal medicine teams, the diagnosis of idiopathic HES was established based on three criteria: marked hypereosinophilia (absolute eosinophil count 9.0 × 10⁹/L), eosinophil-mediated cardiac involvement characterized by isolated right ventricular endomyocardial fibrosis with an associated intracavitary thrombus demonstrated by multimodality imaging, and comprehensive exclusion of secondary, autoimmune, infectious, neoplastic, and clonal causes of eosinophilia.

Treatment was initiated with oral prednisone at a dose of 1 mg/kg/day as first-line therapy to suppress eosinophilic inflammation, together with vitamin K antagonist anticoagulation because of the right ventricular intracavitary thrombus. Intravenous furosemide was also administered to relieve systemic venous congestion and bilateral lower-limb edema. At the time of manuscript preparation, treatment had only recently been initiated, precluding assessment of the clinical response or long-term outcome.

## Discussion

The present case illustrates several important clinical aspects of eosinophilic cardiac disease. First, isolated right ventricular endomyocardial fibrosis is an exceptionally rare manifestation of HES and may closely mimic other intracardiac masses, making the diagnosis particularly challenging [5-7]. In our patient, the coexistence of extensive right ventricular endomyocardial fibrosis and an associated intracavitary thrombus represented an unusual presentation that required a comprehensive multimodality imaging approach for accurate tissue characterization and exclusion of alternative diagnoses. As summarized in Table 2, only a few cases of isolated or predominant right ventricular eosinophilic endomyocardial disease have been reported in the literature. Despite differences in disease stage, all reported cases shared marked eosinophilia, right ventricular apical involvement, and the pivotal contribution of multimodality imaging, particularly cardiac MRI, in confirming endomyocardial fibrosis and differentiating intracavitary thrombus from other right ventricular masses [5-7].

**Table 2:** Previously published cases of isolated or predominant right ventricular eosinophilic endomyocardial disease. Published cases of isolated or predominant right ventricular eosinophilic endomyocardial disease identified in the literature. The table summarizes patient demographics, underlying diagnosis, extent of right ventricular involvement, multimodality imaging findings, therapeutic management, and clinical outcomes. TTE was the initial imaging modality in all reported cases, while CMR played a pivotal role in confirming endomyocardial fibrosis, characterizing intracavitary thrombi, and excluding alternative diagnoses. CMR, cardiac magnetic resonance; HES, hypereosinophilic syndrome; RV, right ventricle; TTE, transthoracic echocardiography

Author	Year	Age/sex	Diagnosis	Right ventricular involvement	Imaging modalities	Treatment	Outcome
Beedupalli and Modi [5]	2016	55/M	Idiopathic HES	Near-complete right ventricular cavity obliteration (early Löffler endocarditis)	TTE, CT, endomyocardial biopsy	Corticosteroids	Complete clinical and hematological remission
Alam et al. [6]	2017	60/F	Eosinophilic endomyocardial fibrosis	Isolated right ventricular endomyocardial fibrosis with restrictive physiology	TTE, CMR	Corticosteroids + surgery	Clinical improvement
Arustamyan et al. [7]	2020	43/M	Idiopathic HES	Right ventricular eosinophilic endocarditis with apical thrombus	TTE, contrast echocardiography, CMR	Corticosteroids + anticoagulation	Complete thrombus regression

One of the major strengths of the present case is the integrated multimodality imaging approach, which proved fundamental for both diagnosis and disease characterization. Transthoracic echocardiography served as the initial imaging modality, revealing an extensive right ventricular intracavitary mass associated with near-complete apical obliteration, marked right atrial enlargement, and tricuspid valve involvement. While echocardiography provides an excellent first-line assessment of cardiac morphology and hemodynamic consequences, its ability to differentiate thrombus from primary cardiac tumors or infiltrative myocardial disorders remains limited [8].

CMR subsequently played the pivotal role in establishing the diagnosis by demonstrating diffuse circumferential subendocardial LGE involving the right ventricular apex and inflow tract, together with an avascular intracavitary thrombus. This characteristic imaging pattern is highly suggestive of eosinophilic endomyocardial fibrosis and enables accurate differentiation from cardiac neoplasms, hypertrophic cardiomyopathy, arrhythmogenic right ventricular cardiomyopathy, and other infiltrative or restrictive cardiomyopathies [9,10]. In addition, CMR precisely delineates the extent of endomyocardial fibrosis and detects myocardial involvement at an earlier stage than conventional echocardiography, making it the reference imaging modality for eosinophilic heart disease [9,10].

CT provided valuable complementary information by confirming the right ventricular filling defect, excluding pulmonary embolism despite the presence of right-sided heart failure, and ruling out thoracic or abdominal malignancy as a potential secondary cause of hypereosinophilia. Therefore, rather than representing competing diagnostic techniques, echocardiography, CMR, and CT should be regarded as complementary imaging modalities, each providing unique anatomical and functional information that collectively increases diagnostic confidence and facilitates comprehensive patient assessment [8,9].

Another noteworthy aspect of this case is the exhaustive etiological evaluation performed before establishing the diagnosis of idiopathic HES. Current international recommendations emphasize that idiopathic HES should only be diagnosed after meticulous exclusion of secondary causes of eosinophilia, including parasitic infections, allergic diseases, autoimmune disorders, vasculitides, solid tumors, hematologic malignancies, and clonal eosinophilic syndromes [1,11,12]. In addition, molecular testing for FIP1L1-PDGFRA, PDGFRB, FGFR1, JAK2, CALR, MPL, and BCR::ABL1 has become an essential component of the diagnostic workup because the identification of an underlying clonal abnormality has important therapeutic and prognostic implications [1,12].

Therapeutic management of eosinophilic cardiac disease is primarily aimed at suppressing eosinophilic inflammation before irreversible myocardial fibrosis develops. High-dose corticosteroids remain the recommended first-line treatment for idiopathic HES and are generally associated with rapid normalization of eosinophil counts and stabilization of cardiac injury [2,4,12]. Anticoagulation is indicated in the presence of intracardiac thrombi because thromboembolic complications represent a major source of morbidity and mortality in patients with eosinophilic heart disease [2,4].

Cardiac involvement remains the principal determinant of long-term prognosis in HES [2,3]. Although medical therapy effectively controls eosinophilic activity in most patients, established endomyocardial fibrosis may progress to restrictive cardiomyopathy, chronic right-sided heart failure, recurrent intracardiac thrombosis, thromboembolic events, ventricular arrhythmias, and significant valvular dysfunction [2,3]. In the present case, corticosteroid therapy and anticoagulation were initiated promptly after the diagnosis of idiopathic HES with isolated right ventricular endomyocardial fibrosis and intracavitary thrombus. Nevertheless, long-term clinical and multimodality imaging follow-up remains essential to evaluate treatment response, monitor thrombus resolution, and detect disease progression or relapse. Consequently, early recognition through multimodality cardiovascular imaging is essential, as prompt diagnosis and treatment before irreversible fibrotic remodeling develops are associated with significantly improved clinical outcomes [1,9].

Taken together, this case expands the limited body of literature describing isolated right ventricular eosinophilic endomyocardial fibrosis with intracavitary thrombus. More importantly, it underscores the pivotal value of a multimodality imaging strategy, in which echocardiography provides the initial diagnostic suspicion, CMR offers definitive tissue characterization and disease staging, and CT excludes alternative thoracic and extracardiac pathologies. This integrated diagnostic approach is essential for distinguishing this rare entity from other causes of right ventricular masses and enables timely initiation of disease-modifying therapy before irreversible myocardial damage occurs.

## Conclusions

Idiopathic HES should be considered in patients presenting with unexplained hypereosinophilia and an intracardiac mass, even in the absence of typical systemic manifestations. Isolated right ventricular endomyocardial fibrosis with intracavitary thrombus is an exceptionally rare presentation that poses significant diagnostic challenges because it may mimic other intracardiac pathologies. This case highlights the complementary role of multimodality cardiovascular imaging, particularly transthoracic echocardiography, CMR, and CT, in accurately characterizing the lesion, excluding alternative diagnoses, and guiding clinical management. Early recognition, together with a comprehensive etiological evaluation and timely initiation of appropriate therapy, is essential to prevent irreversible cardiac remodeling and improve long-term outcomes.
